# Years of potential life lost due to AIDS in female, in Southern Brazil: a descriptive study, 2007-2017

**DOI:** 10.1590/S2237-96222022000300010

**Published:** 2022-12-02

**Authors:** Maiton Bernardelli, Douglas Nunes Stahnke, Marcos Pascoal Pattussi, Laura Cecilia López, Tonantzin Ribeiro Gonçalves

**Affiliations:** 1 Centro Universitário da Serra Gaúcha, Caxias do Sul, RS, Brazil; 2 Universidade do Vale do Rio dos Sinos, Programa de Pós-Graduação em Saúde Coletiva, São Leopoldo, RS, Brazil

**Keywords:** AIDS, Life Expectancy, Women, Social Vulnerability, Racial Groups, Descriptive Epidemiology

## Abstract

**Objective:**

to describe the years of potential life lost (YPLL) due to AIDS among the female population and analyze its association with race/skin color and social vulnerability indicators in Porto Alegre, capital city of the state of Rio Grande do Sul, Brazil.

**Methods:**

this was a descriptive study that took into consideration AIDS deaths in female between 2007 and 2017; data were obtained from the Mortality Information System; crude values and YPLL rates per 1,000 deaths were calculated, taking into consideration health districts and race/skin color.

**Results:**

of the 1,539 deaths, approximately 51,000 years of potential life were estimated, representing 86.5 years lost/1,000 female; it could be seen a higher proportion of deaths among female of White race/ skin color (53.4%); however, a higher rate of YPLL was found among female of Black and mixed race/skin color living in regions of greater vulnerability.

**Conclusion:**

the results suggest the impact of racial inequalities on the decrease in years of potential life due to AIDS deaths.

**Study contributions**

**Main results**

Female living with AIDS of Black/mixed-race skin color and/or residing in health districts of greater social vulnerability presented higher rates of YPLL, suggesting the impact of racial inequalities on premature mortality in the context of AIDS.

**Implications for services**

Health services should be aware of social and racial inequalities in the care provided to the population living with HIV, highlighting the need for their actions to be effective in preventing deaths among young female.

**Perspectives**

To promote actions that focus on comprehensive health care for Black female in vulnerable situations, not only related to sexual and reproductive health, especially those that provide care for the Black population in greater social vulnerability.

## Introduction

Globally, AIDS-related diseases are the leading causes of death among female of reproductive age, or pregnant and puerperal female.^1^ In Brazil, 49% of deaths among females in 2017 occurred in the 25 to 39 age group,^2^ resulting in years of potential life lost among these female.

In 2018, in Porto Alegre, capital city of the state of Rio Grande do Sul, an AIDS-related mortality rate of 24 deaths per 100,000 inhabitants was recorded, surpassing the national indicator by five times.^2^ Since 2007, Porto Alegre has shown a persistent increase in AIDS-related mortality rates among female,^3^ ^,^ ^4^ ranking among the capital cities with the highest rates, especially among female aged 30 to 39 years old.^5^

Despite the evidence of a high burden of AIDS-related diseases in female,^1^ there are few studies dedicated to investigating the years of potential life lost (YPLL) in this segment of the population,^6^ ^-^ ^9^ which are studies aimed to investigate the number of years that a given population who die prematurely, for a given cause, have not lived.^10^ One of them, conducted in Tanzania, on causes of premature mortality, showed that AIDS accounted for the highest number of YPLL, including an increase in this indicator between 2006 and 2015, and that female had more YPLL for this cause than men.^6^ In Latvia, one of the Eastern European countries with the highest AIDS mortality rates, an investigation of YPLL between 1991 and 2001 did not identify differences between men and female or a higher rate among injecting drug users and immigrants.^7^ In Brazil, a study analyzing YPLL due to AIDS, for the period 1985-2006, explored the association of premature mortality with social vulnerability indicators and low level of education in female living in the state of São Paulo, and did not find enough evidence to suggest that vulnerabilities have an impact on the reduction of years of potential life among this population.^8^ In Pernambuco, a study on YPLL due to AIDS between 1996 and 2005 showed that the increase in the years of potential life lost due to AIDS results from the expansion of the epidemic in regions of greater urbanization, where inequality in access to health services and the social determinants may influence the indicator.^9^

The analysis of YPLL by capturing premature mortality more accurately, as well as related economic and social inequalities, can contribute to the evaluation of the conditions and health status of the population.

The aim of this study was to describe the YPLL due to AIDS in the female population living in Porto Alegre, state of Rio Grande do Sul, Brazil, and analyze its possible association with social vulnerability indicators, according to the health districts of the largest capital city in Southern Brazil. This study was published in preprint version.^11^

## Methods

### Study design

This was a descriptive study, based on data from the Mortality Information System (*Sistema de Informações sobre Mortalidade* - SIM) of epidemiological surveillance in Porto Alegre.

### Setting

Porto Alegre, the capital city of the state of Rio Grande do Sul, according to the 2010 Population Census, had 1,409,351 inhabitants, with a predominance of females: 53% (755,564).^12^ The city has presented a persistent increase in AIDS-related mortality rate among female since 2007, reflected in a rate that makes it stand out among other Brazilian capitals.^2^ The structure of health services in Porto Alegre, in 2017, was comprised of 146 primary healthcare centers (PHC), four specialized care services for people living with HIV/AIDS and a counseling and testing center for the general population.^13^ As units of analysis of the study, the 17 health districts of the municipality were taken into consideration: Ilhas, Humaitá-Navegantes, Centro, Noroeste, Norte, Eixo Baltazar, Eixo Leste, Nordeste, Glória, Cruzeiro, Cristal, Sul, Centro-Sul, Partenon, Lomba do Pinheiro, Restinga and Extremo Sul.

### Data source and measurement

Death data were retrieved from the SIM database, provided by epidemiological surveillance - Health Surveillance Department/Municipal Health Secretariat of Porto Alegre. Information on race/skin color (White or Black) was retrieved from Death Certificate (DC) records. We took into consideration deaths whose underlying cause was registered as AIDS-related cause, according to code B20-24, defined by the International Statistical Classification of Diseases and Related Health Problems 10^th^ Revision (ICD-10: B20-24). Information on the population living in the municipality and by health district was obtained from surveys conducted by the Brazilian Institute of Geography and Statistics (*Instituto Brasileiro de Geografia e Estatística* - IBGE) for the year 2010.^12^

### Participants

We analyzed the death records of the female population aged between 15 and 75 years old, living in Porto Alegre, who had AIDS as the underlying cause, between 2007 and 2017. Life expectancy at birth of 77.6 years (2010) was taken into consideration.^12^ The definition of 75 years of age as the upper limit to human lifespan was taken into consideration because this value is close to the estimated life expectancy at birth, excluding those over 75 years old.^4^ ^,^ ^8^ ^,^ ^9^ The study addressed the female population over 15 years of age; and those younger than this age were excluded because the indicator is not sensitive to this age group.^14^

### Variables

The study variables were: social vulnerability index (SVI) of the health district (HD); characterization of cases (age group; race/skin color); and YPLL taking into consideration the analysis period (2007-2017). The categories of White race/skin color and Black/mixed race/skin color were taken into consideration, the latter resulting from the grouping of the two categories, according to IBGE recommendations.^15^ Ages were grouped by five-year age groups (15-19; 20-24; 25-29; 30-34; 35-39; 40-44; 45-49; 50-54; 55-59; 60-64; 65-69; 70-75). The SVI of each HD was calculated by adopting the methodology of the Institute for Applied Economic Research (*Instituto de Pesquisa Econômica Aplicada* - IPEA),^16^ based on 16 indicators described in the Atlas of Social Vulnerability for Brazilian Municipalities, encompassing the following dimensions: urban infrastructure; human capital; income and work. Each dimension is comprised of indicators that receive weighted mean, and the arithmetic mean of the three dimensions comprises the SVI.^16^ For the HD in Porto Alegre, the following values were considered: "low vulnerability", values from 0.000 to 0.200; "medium vulnerability", values from 0.201 to 0.300; and "high vulnerability", values from 0.301 to 0.500. YPLL rates per 1,000 females were calculated according to the HD where the death occurred, divided by the female population residing in the same HD, in the age group studied.

### Bias control

Taking into consideration that two populations with different causes of mortality may generate absolute numbers of similar YPLL, even though they have different population sizes, we calculated both the absolute number and the relative number of YPLL, represented by their rate, aiming to obtain a more complete picture of the outcome investigated. In order to make comparisons between the units of analysis, in different years, age-standardized YPLL rates were used, thus reducing the influence of different age structures.^17^

### Statistical methods

The absolute and relative frequencies were measured taking into consideration the variables of interest (SVI, race/skin color and YPLL). The calculation of the absolute value of the YPLL of each period studied (2007; 2017; 2007 to 2017) was performed by multiplying the number of deaths in each age group by the number of remaining years of life, considering 75 years of age as the upper limit. The total number of YPLL was obtained by adding the YPLL in each age group, applying the following formula:

YPLL=∑ai x di

where *ai* represents the difference between the age limit (75 years old) and the midpoint of age in each age group (2.5), assuming a uniform distribution of deaths in each group; and *di* is the number of AIDS deaths in the same age group.

To calculate the rates of YPLL per 1,000 females, we used the ratio obtained by adding the YPLL per age group divided by the total number of inhabitants in the same age group multiplied by 1,000. The average number of YPLL was calculated as the result of dividing the total number of YPLL by the number of deaths analyzed, in order to know the average age at which the deaths occurred. Then, to calculate the same indicators according to the HD, we took into consideration the population and the number of deaths of each health district in the years investigated.

Pearson's correlation analysis was performed to assess possible associations of YPLL rates with (i) levels of social vulnerability index and (ii) the proportion of Black female in the HDs. Data tabulation and the calculation of indicator were performed using the Microsoft Excel and Statistical Package for the Social Sciences (SPSS 2.0) applications.

### Ethical aspects

This paper is part of a larger study entitled *Space-time indicators and risk factors associated with mortality in female living with HIV*, approved by the Research Ethics Committees of the Universidade do Vale do Rio dos Sinos [CEP/Unisinos: Opinion No. 3,233,242, approved on March 29, 2019; Certificate of Submission for Ethical Appraisal (CAAE) No. 06210919.7.0000.5344] and the Municipal Health Department of Porto Alegre (CEP/SMSPA: Opinion No. 3,281,948, approved on April 24, 2019; 06210919.7.3001.5338).

## Results

**Figure 1:**
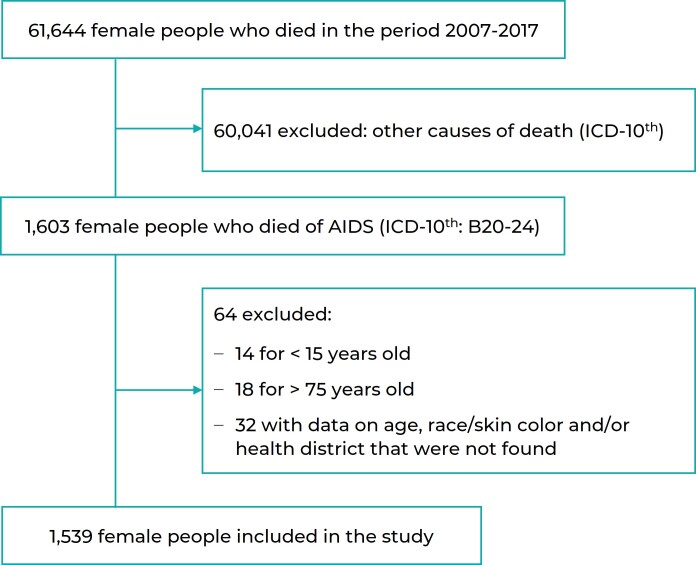
Selection process of AIDS deaths in the female population living in Porto Alegre, capital city of the state of Rio Grande do Sul, Brazil, 2007-2017

Between 2007 and 2017, 1,603 deaths among females living in Porto Alegre whose underlying cause was AIDS, were registered. We excluded 14 records of people under the age of 15, and 18 records of those over 75 years of age; and a further 4 records of female of Asian and Indigenous race/skin color. Exclusions for missing data related to age, race/skin color and health district totaled 32. The final sample was comprised of 1,539 death records (Figure 1).

AIDS deaths were more frequent in the female population aged 40 to 44 years (n = 259; 16.8%), of White race/skin color (n = 839; 54.5%). Female of Black and mixed race/skin color accounted for 45.5% (n = 700). For the period, 51,075 YPLL due to AIDS were estimated in the study population. The rate of YPLL due to AIDS was 86.5 per 1,000 females - an average of 32.5 YPLL. When compared to White female, with 54.4 YPLL/1,000 (an average of 32.0 YPLL), Black/mixed-race female presented a higher YPLL rate, 200.3 YPLL/1,000 (an average of 33.4 YPLL) (Table 1 and Table 2).

**Table 1:** Absolute number, percentage, rate and average of years of potential life lost due to AIDS in female people (n = 1,539) living in Porto Alegre, Rio Grande do Sul, Brazil, 2007-2017

Characteristics	Total (2007-2017)					2007					2017			
	Deaths (%)	YPLL^a^	YPLL^a^ rate	YPLL^a^ average		Deaths (%)	YPLL^a^	YPLL^a^ rate	YPLL^a^ average		Deaths (%)	YPLL^a^	YPLL^a^ rate	YPLL^a^ average
**Age group**														
15-19	15 (1.0)	862.5	16.5	57.5		-	-	-	-		2 (1.6)	115.0	2.2	57.5
20-24	65 (4.2)	3,570.0	58.9	52.5		2 (1.3)	105.0	1.7	52.5		2 (1.6)	105.0	1.7	52.5
25-29	149 (9.5)	7,077.5	104.2	47.5		24 (15.8)	1,140.0	16.8	47.5		8 (6.2)	380.0	5.6	47.5
30-34	225 (14.5)	9,647.5	155.7	42.5		28 (18.4)	1,190.0	19.2	42.5		12 (9.3)	510.0	8.2	42.5
35-39	246 (15.9)	9,337.5	180.7	37.5		21 (13.8)	787.5	15.2	37.5		19 (14.7)	712.5	13.8	37.5
40-44	259 (16.8)	8,677.5	172.3	32.5		33 (21.7)	1,072.5	21.3	32.5		26 (20.2)	845.0	16.8	32.5
45-49	184 (11.9)	5,115.0	92.0	27.5		20 (13.2)	550.0	9.9	27.5		20 (15.5)	550.0	9.9	27.5
50-54	163 (10.7)	3,780.0	70.8	22.5		10 (6.6)	225.0	4.2	22.5		15 (11.6)	337.5	6.3	22.5
55-59	99 (6.4)	1,767.5	37.9	17.5		8 (5.3)	140.0	3.0	17.5		7 (5.4)	122.5	2.6	17.5
60-64	63 (4.1)	812.5	21.4	12.5		4 (2.6)	50.0	1.3	12.5		9 (7.0)	112.5	3.0	12.5
65-69	46 (3.1)	360.0	12.6	7.5		1 (0.7)	7.5	0.3	7.5		4 (3.1)	30.0	1.1	7.5
70-75	25 (1.7)	67.5	2.9	2.5		1 (0.7)	2.5	0.1	2.5		5 (3.9)	12.5	0.5	2.5
Total	1,539 (100.0)	51,075.0	86.5	32.5		152 (100.0)	5,270.0	8.9	34.7		129 (100.0)	3,832.5	6.5	29.7
**Race/skin color**														
White	53.4	26,842.5	54.4	32.0		57.2	2,862.5	5.8	32.9		45.0	1,570.0	3.2	27.1
Black/mixed-race	446	23,370.0	200.3	33.4		41.5	2,342.5	20.1	37.2		51.9	2,142.5	18.4	32.0

a) YPLL: Years of potential life lost; b) SVI: Social vulnerability index.

It could be seen a decrease in the average number of YPLL between the first (2007) and the last year (2017) of the series studied. In 2007, the average age of death was 40.3 years old, rising to 45.3 years old in 2017. Regarding the differences in the indicator by race/skin color, there was also an increase in the average age of death: in 2017, while the average age of death was 43 years old among Black/mixed-race female, the average age of death among White female was 48 years old (Table 2).

**Table 2:** Distribution of deaths and average age (in years, per 1,000 female) in AIDS death among female (n = 1,539) living in Porto Alegre, Rio Grande do Sul, Brazil, 2007-2017

Race/skin color	Deaths n (%)			Average age of deaths		
	2007-2017	2007	2017	2007-2017	2007	2017
White	839 (54.5)	87 (57.2)	58 (45.0)	43.0	42.1	48.0
Black/mixed race	700 (45.5)	65 (42.8)	71 (55.0)	41.6	37.8	43.0
Total	1,539 (100.0)	152 (9.9)	129 (8.4)	42.5	40,.3	45.3

As for the analysis of YPLL according to HD, it could be seen the highest rates in the following HDs: Cruzeiro (220.9 YPLL/1,000 female), Lomba do Pinheiro (175.5 YPLL/1,000 female) and Restinga (168.3 YPLL/1,000 female). In these regions, the average age of death was 42 years old. The HDs in Ilhas (0.48), Nordeste (0.33), Lomba do Pinheiro (0.31) and Restinga (0.31) showed the highest SVI, and their values for YPLL were higher when compared to YPLL in the HDs with lower SVI (Table 3). Pearson's bivariate analysis identified a weak correlation between the YPLL rates of the HDs and the SVI levels (r = 0.557; p-value = 0.020), as well as between YPLL rates and the proportion of Black/mixed-race female living in each HD (r = 0.560; p-value = 0.020). An inverse relationship was observed between YPLL rates and the proportion of White female (Table 4).

**Table 3:** AIDS deaths (n), average age of deaths, proportion of deaths according to White and Black/mixed race/skin color, average rate of years of potential life lost due to AIDS among the female population and social vulnerability index score, according to health districts, Porto Alegre, Rio Grande do Sul, Brazil, 2007-2017

Health Districts	AIDS deaths among famales				Average rate of YPLL^a^ (per 1,000 female)	SVI^b^
	n	Average age of deaths (years)	White race/skin color (%)	Black/mixed race/skin color (%)		
Cruzeiro	155	41.0	53.2	46.8	220.9	0.27
Lomba do Pinheiro	114	42.0	54.0	46.0	175.5	0.31
Restinga	115	42.2	42.1	57.9	168.3	0.31
Nordeste	51	43.9	46.2	53.8	127.9	0.33
Glória	77	40.9	56.6	43.4	121.0	0.27
Humaitá/Navegantes	69	42.8	54.4	45.6	120.2	0.25
Leste	163	42.1	48.2	51.8	119.3	0.25
Eixo Baltazar	110	44.1	60.9	39.1	86.8	0.15
Extremo Sul	32	41.4	76.7	23.3	84.3	0.27
Ilhas	8	45.0	87.5	12.5	84.1	0.48
Norte	96	42.2	55.2	44.8	77.4	0.26
Centro-Sul	89	42.7	65.9	34.1	69.4	0.22
Cristal	22	39.3	70.8	29.2	60.4	0.22
Sul	52	42.6	77.4	22.6	46.1	0.22
Partenon	174	44.1	48.6	51.4	41.4	0.25
Centro	136	43.0	56.2	43.8	34.4	0.17
Noroeste	48	44.3	65.3	34.7	25.2	0.18
Total	1,511	32.5	54.5	45.5	86.5	-

a) YPLL: Years of potential life lost; b) SVI: Social vulnerability index.

**Table 4:** Correlation coefficients between rates of years of potential life lost due to AIDS in the female population, social vulnerability index and population proportion by race/skin color, Porto Alegre, Rio Grande do Sul, Brazil, 2007-2017

Variable	YPLL^a^ r(95%CI)^b^	p-value
SVI^c^	0.557 (0.154;0.874)	0.020
White female (%)	-0.560 (- 0.812; -0.347)	0.020
Black/mixed-race female (%)	0.560 (0.350;0.796)	0.020

a) YPLL: Years of potential life lost; b) 95%CI: 95% confidence interval; c) SVI: Social vulnerability index.

**Supplementary Figure 1:**
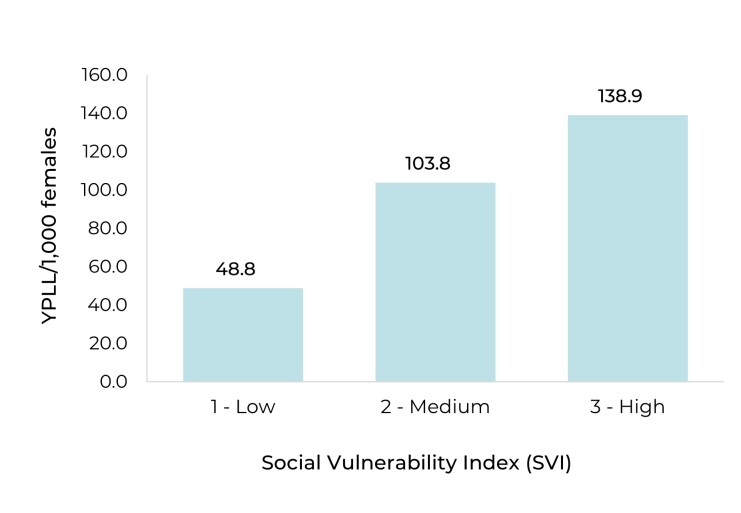
Rate of years of potential life lost due to AIDS according to the social vulnerability index of health districts, Porto Alegre, Rio Grande do Sul, Brazil, 2007-2017

When the HDs were grouped according to SVI levels, it could be seen that the average rates of YPLL due to AIDS, in the category of health district with the lowest SVI level, was 48.8 years lost per 1,000 females, while the average rate of YPLL in the HD group with medium social vulnerability was 103.8 years lost/1,000, and in the HD group with the highest SVI, it was 138.9 YPLL/1,000 female. The average rate of YPLL in the category of districts with high social vulnerability was 185% higher than the average rate of YPLL observed in the category of districts with a lower SVI level (Supplementary Figure 1).

## Discussion

The study showed a reduction in YPLL due to AIDS between the first and last year of the series studied and, consequently, an increase in the average age at death. However, the average age of death due to AIDS was lower among Black/mixed-race female living in health districts of higher vulnerability.

While the increase in the average age of deaths corroborates the results achieved with the universal access to antiretroviral therapy in Brazil, which has led to a decrease in AIDS deaths, this study shows that the impact of this measure was different on Black/mixed-race female, possibly due to social inequalities and care failures.^18^ It is noteworthy that, even with the advances in public policies in the field of HIV/AIDS, the lack of access to health services, especially in regions of greater social vulnerability, where living conditions are precarious, illness and death causes are potentiated.^19^

It is necessary to consider the existence of some limitations of this study. For example, the exclusion of deaths among female aged under 15 years old and over 75 years old in the calculation of YPLL may lead to underestimation of this indicator. Regardless of the low number of exclusions, specific studies, focused on these age groups and addressing these gaps, are suggested. Another limitation of this study lies in the AIDS deaths underestimated in the SIM database, because, in addition to the stigma that generates underreporting, there is a significant number of external causes (suicide, feminicide, among others) that can hide deaths. It is worth highlighting that, although a higher prevalence of deaths among White female was identified, this could reflect the population structure of the municipality, with approximately 79% self-declared White residents according to the 2010 Census.^12^ Moreover, it is worth mentioning that as a result of structural racism, Black people may be more likely to have an AIDS mortality record than White people in the same condition,^20^ contributing to the invisibility of health actions aimed at Black people, especially in HIV/AIDS prevention and care strategies.

It could be seen a possible impact of the expressive social gradient on health conditions of the female who were investigated, i.e.: the worse the social conditions of certain health districts, the higher the YPLL rates. In the health districts of Cruzeiro, Lomba do Pinheiro and Restinga, the high rates of social vulnerability reflect the lack of resources and basic sanitation, and these three districts concentrate most of the Black population of the city. This fact was also observed among female from the state of São Paulo, especially young adults, with low level of education, drug users and living in regions of higher vulnerability.^8^ These findings highlight the presence of systematic inequalities, which potentiate early deaths due to AIDS.^3^

Thus, premature death among Black/mixed-race female may reflect processes of structural racism that normalize the distribution of privileges/disadvantages among different racial groups, resulting in inequalities in access to diagnosis and healthcare services, as well as in living conditions, illness and death among these female.^21^ An analysis of YPLL due to AIDS in the United States observed evidence of inequalities in early mortality, and the impact on the average age of death was significantly higher for Black/mixed-race female than for White female.^22^

The scenario of inequalities for the Black population in Brazil is linked to structural racism, which determines the worst social and health indicators by generating disadvantages as a normalized social form.^23^ Black and mixed-race female experience vulnerabilities that intersect gender and social class,^24^ ^,^ ^25^ given that when compared to White female, most Black female also belong to the group with the lowest level of education and income, live in more precarious housing conditions and they are, more often, the head of household^26^ and therefore, more exposed to different types of violence.^27^

Regarding the utilization of sexual and reproductive health services in the country, Black/mixed-race female are the most exposed to individual and institutional barriers to accessing care, from seeking the service to the moment of care,^28^ and they are also the ones who suffer most from serious neglect, on the point of leading to death.^29^ In the field of HIV/AIDS, a comparative study of Black and White female living with HIV in São Paulo, capital city of the state of São Paulo, pointed out several differences that negatively affected Black female and impacted on the quality of care received.^27^

Although the economic factor was not evaluated in this study, financial barriers may impact indicators such as YPLL. Black and mixed-race female are the most exposed to lack of resources for transportation, including access to health care via the SUS, which is reflected in the health inequalities observed in the country.^30^ These observations reinforce the contextual nature of vulnerabilities to HIV/AIDS, and these characteristics should be taken into consideration when structuring the care provided to female living with HIV, especially those most affected by social inequalities.

The findings of this study suggest the need to develop care actions and strategies aimed at preventing these deaths, especially among female. The results also warn of the high social cost of early deaths, which can potentiate vulnerabilities toward children and family members, in addition to violating the human right to life of these female. Given the complexity of the factors involved, it is important to increase efforts to minimize structural vulnerabilities related to race/skin color, sex and social class, in order to reduce AIDS deaths and their impact on the worsening health status of the female population in Brazil.
